# A single-arm phase Ib study of personalized peptide-pulsed dendritic cell-based multiple target cytotoxic T lymphocyte immunotherapy in combination with toripalimab as second-line therapy in advanced non-small-cell lung cancer

**DOI:** 10.1007/s00262-026-04338-7

**Published:** 2026-03-11

**Authors:** Weihong Zhang, Jing Qin, Jing Luo, Yanjuan Xiong, Fan Yang, Bin Li, Meng Shen, Huizhen Geng, Zibo Li, Xiao Tian, Shuzhan Li, Runmei Li, Yang Wang, Xinwei Zhang, Xiubao Ren, Qian Sun, Li Zhou, Liang Liu

**Affiliations:** 1 Department of Immunology and Biotherapy, Key Laboratory of Cancer Immunology and Biotherapy, Key Laboratory of Cancer Prevention and Therapy, Tianjin’s Clinical Research Center for Cancer, Tianjin Medical University Cancer Institute and Hospital, National Clinical Research Center for Cancer, Tianjin, 300060 China; 2 Hebei Bio-High Technology Development Co., Ltd., Shijiazhuang, Hebei China

**Keywords:** Lung cancer, Multiple target cytotoxic T lymphocyte, Dendritic cell, Combination therapy, PD-1

## Abstract

**Background:**

Anti-programmed cell death 1 (PD-1)/programmed death-ligand 1 (PD-L1) monoclonal antibody therapy has been approved as second-line treatment for advanced non-small-cell lung cancer (NSCLC). Unfortunately, the objective response rate (ORR) is not satisfied. T lymphocytes obtained by co-culture with personalized tumor-specific antigenic peptide-pulsed dendritic cells (DC), also known as multiple target cytotoxic T lymphocytes (MCTL) can restore the antitumor immunity and potentially improve clinical outcomes. We conducted a clinical study evaluating MCTL immunotherapy combined with toripalimab in patients with advanced NSCLC.

**Methods:**

This single-center, open-label, phase Ib trial (NCT04193098) evaluated the combination of MCTL and toripalimab as second-line therapy in 21 patients with advanced NSCLC. Peripheral blood samples were collected for antigenic peptide analysis, and patient immune status was assessed. The primary and secondary endpoints were to evaluate safety and clinical outcomes, respectively.

**Results:**

Among the 21 patients, the ORR and disease control rate (DCR) were 33.3 and 76.2%, respectively. Median overall survival (mOS) was 24.1 months, and median progression-free survival (mPFS) was 8.6 months. Most patients demonstrated improved immune status post-treatment compared to baseline. Patients exhibiting pronounced immune enhancement following MCTL/toripalimab therapy tended to have better clinical outcomes. No significant adverse events (AEs) were observed during combination therapy.

**Conclusion:**

In this cohort of patients with advanced NSCLC, MCTL/toripalimab therapy demonstrated manageable safety and promising antitumor efficacy as a second-line treatment. Further studies are warranted to confirm these results.

**Supplementary Information:**

The online version contains supplementary material available at 10.1007/s00262-026-04338-7.

## Introduction

Lung cancer remains the leading cause of cancer-related mortality in China, with non-small-cell lung cancer (NSCLC) accounting for nearly 80% of all cases [1–3]. Although surgery offers a potential cure for early-stage disease, most patients present with advanced NSCLC, where immunotherapy has become increasingly important. Over the past decade, immune checkpoint inhibitors (ICIs), particularly anti–programmed cell death-1 (αPD-1) and anti–programmed death-ligand 1 (αPD-L1) antibodies, have revolutionized treatment and significantly improved survival outcomes [4]. However, only about 20% of patients respond to monotherapy [5, 6], underscoring the urgent need for novel strategies to enhance ICIs efficacy.

Adoptive cell therapy (ACT) is a promising immunotherapeutic approach that amplifies antigen-specific immune responses by reinfusing autologous immune cells expanded ex vivo [7, 8]. While antigen-specific modalities such as chimeric antigen receptor (CAR)-T cell therapy have shown efficacy in hematologic malignancies, their success in solid tumors remains limited [9]. Recently, dendritic cell (DC)–based cytotoxic T lymphocyte (CTL) therapies have demonstrated the potential to enhance antitumor immunity by activating both innate and adaptive responses [10, 11]. Multiple target cytotoxic T lymphocyte (MCTL) therapy, generated by co-culturing patient-derived T cells with personalized tumor peptide–pulsed DCs, may overcome previous limitations. By combining MCTL with PD-1 blockade, such as toripalimab, the antitumor immune response could be further strengthened, providing a promising new therapeutic strategy for advanced NSCLC.

Here we report the first clinical trial evaluating the combination of MCTL with αPD-1 antibody Toripalimab in advanced NSCLC patients (NCT04193098). In addition to addressing the feasibility, safety and efficacy of this combination therapy, we conducted comprehensive molecular and immunological analyses to explore potential mechanisms. The combination therapy described in this study offers a novel therapeutic approach for advanced NSCLC by enhancing neoantigen-specific T cell responses to achieve effective tumor control.

## Methods

### Study design

This study (ClinicalTrials.gov: NCT04193098) was an open-label,

single-center phase Ib trial that enrolled 21 patients at Tianjin Medical University Cancer Institute and Hospital between June 2019 and August 2021. The study was conducted in accordance with the Declaration of Helsinki and the principles of Good Clinical Practice. The protocol was approved by the Institutional Review Board, and all patients provided written informed consent before the study procedures. The eligibility criteria included: (I) 18–75 years of age; (II) histologically or cytologically confirmed stage III-IV NSCLC; (III) receipt of one cycle of prior platinum-based chemotherapy; (IV) tyrosine kinase inhibitor (TKI) therapy permitted for patients with known ALK translocations or EGFR mutations. Blood samples were collected from all patients before and after treatment for biomarker analysis.

### Treatments and follow-up

**Fig. 1:**
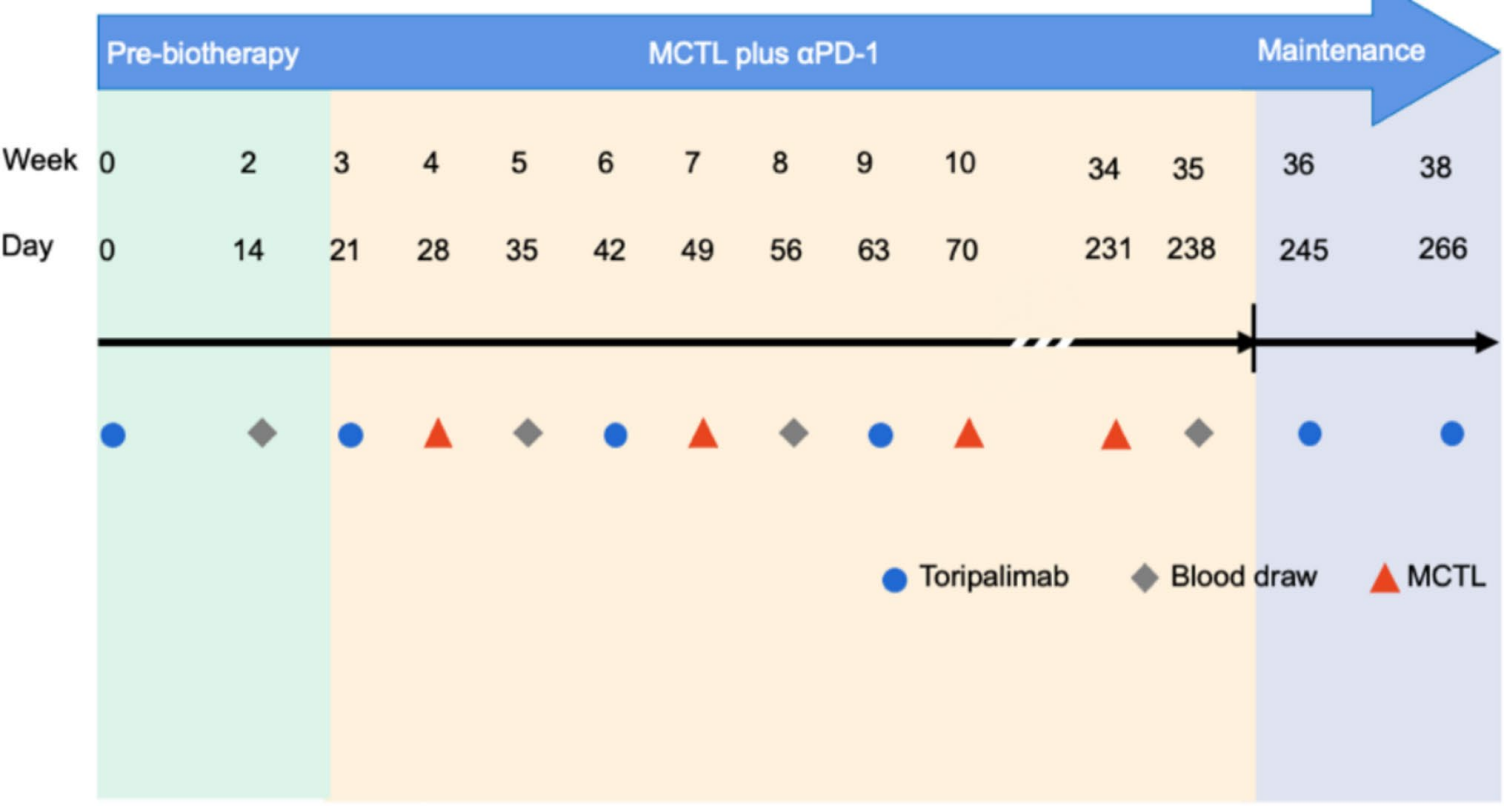
MCTL combined with Toripalimab clinical study design. Patients treated with Toripalimab was initiated at week 0 and every 3 weeks for one cycle. MCTL (10^9^ cells) was administrated started in week 4 and 1 week after Toripalimab. Then use Toripalimab as maintenance treatment. Blood draw and leukapheresis were performed between the administration of Toripalimab and MCTL. MCTL, multiple target cytotoxic T lymphocyte; αPD-1, anti-PD-1

Patients received intravenous administration of Toripalimab 240 mg initiated at week 0 and repeated every 3 weeks per cycle. Intravenous infusion of MCTL (10⁹ cells) commenced at week 4, one week following the initial dose of Toripalimab. Toripalimab was administered up to 12 cycles, followed by maintenance therapy with Toripalimab continued until disease progression or unacceptable toxicity occurred. Blood collection and leukapheresis were conducted between Toripalimab and MCTL administrations at specified time points. The treatment schedule is illustrated in Fig. 1. The primary objective of this study was to evaluate the safety of Toripalimab combined with MCTL and to assess the overall response rate (ORR) according to the Response Evaluation Criteria in Solid Tumors (RECIST) version 1.1 for the combination regimen. Secondary endpoints included progression-free survival (PFS), disease control rate (DCR), and overall survival (OS). Additionally, the antitumor activity of the combination and the immune responses induced by the combined therapy were assessed. Patients were followed up for up to 60 months. Tumor recurrence or metastasis was monitored after treatment completion using ultrasound imaging, computed tomography (CT), positron emission tomography/computed tomography (PET/CT), magnetic resonance imaging (MRI), and bone scintigraphy, with evaluations repeated every 6 months thereafter. Short-term efficacy was classified as complete response (CR), partial response (PR), stable disease (SD), or progressive disease (PD), based on RECIST version 1.1. Lesions deemed non-measurable (e.g., pleural effusions) were not evaluated unless they contributed to disease progression. All treatment-related toxicities were clinically assessed according to the Common Terminology Criteria for Adverse Events (CTCAE) version 5.0 and independently reviewed by two oncologists to determine the type and severity of toxicity.

### Screen and identify personalized tumor-specific antigenic peptides

Peripheral blood (5–8 mL) was collected from patients without anticoagulants and processed for serum isolation. Lipids, high-abundance proteins, and low-molecular-weight metabolites were removed from the serum samples using acetonitrile precipitation followed by solid-phase extraction with hydrophilic–lipophilic balance (HLB) cartridges, yielding enriched target polypeptides. The purified serum peptidome was analyzed via quadrupole time-of-flight mass spectrometry (Q-TOF MS; Waters Acquity UPLC/Xevo G2 QTOF system). Peptide targets identified through MS were cross-referenced with a proprietary MCTL tumor-specific target database (bio-high-technology) to select patient-individualized antigens. All candidate antigenic peptides (Table S1) were chemically synthesized for subsequent use in MCTL culture.

### Generation of mature personalized tumor-specific peptide-pulsed DC

Peripheral blood mononuclear cells (PBMCs) were isolated from 50–60 mL of patient blood using Ficoll density gradient centrifugation, yielding approximately 6 × 10^7^–1.2 × 10^8^ cells. Following standard washing procedures, the PBMC were resuspended in serum-free GT-T551 medium (Takara, Japan) at a 2 × 10^6^ cells/mL density and incubated for 2 h in a cell culture incubator (37 °C, 5% CO_2_). Adherent cells were differentiated into DCs by culturing in GT-T551 medium supplemented with: (1) 500 U/mL recombinant human IL-4 (rhIL-4; CellGenix, Germany); (2) 1000 U/mL recombinant human GM-CSF (rhGM-CSF; Liaoning WX, China); (3) 100 ng/mL TNF-α (R&D Systems, USA); (4) 50 μg/mL personalized tumor-specific antigenic peptides (as previously described). On day 5, mature peptide-pulsed DCs were harvested.

### Culture of MCTL

To expand antigen-specific MCTL, non-adherent PBMC from each patient were cultured in GT-T551 medium supplemented with: (1) 1000 U/mL IFN-γ (Beijing SL, China); (2) 100 ng/mL anti-CD3 antibody (Boehringer Mannheim, Germany); (3) 1000 U/mL recombinant human IL-2 (rhIL-2, Beijing SL, China). After 5 days of stimulation, the cells were co-cultured with mature personalized tumor-specific peptide-pulsed DC population (as described above). The culture medium was replenished every 2–3 days with fresh autologous serum-containing medium and IL-2. Prior to cell infusion, the expanded MCTLs were evaluated for tumor-specific T cell receptor (TCR) positivity ratio.

### Flow cytometry

The proportions of immune cells and cytokine production as well as TCR ratio were measured by flow cytometry. PBMCs and MCTLs from each patient were obtained as described previously. For the staining of single-cell suspensions, all incubations were performed on ice. Cells were initially incubated with Zombie NIR (1/500), diluted in PBS for 30 min to distinguish dead and living cells, then washed twice, incubated with a mixture of antibodies in fluorescence-activated cell sorting (FACS) buffer (2% fetal bovine serum in PBS) for 30 min, washed twice again, and suspended in staining buffer. Intracellular staining for chemokines were performed in Perm/Wash buffer, followed by a washing step, and suspension in FACS buffer. Intranuclear staining of Foxp3 using a Foxp3/Transcription Staining Buffer Set according to the manufacturer’s instructions. Data were collected using BD LSR Fortessa (BD Biosciences, San Jose, CA, USA) flow cytometer and analyzed in FlowJo version 9 (USA).

### TCR detection

Personalized tumor-specific antigenic peptides were obtained as described above and were dissolved in serum-free medium to a final concentration of 5 mmol/mL. The peptide-HLA tetramer complexes (TB-7300-K2, MLB, USA) were assembled by mixing the three components at a molar ratio of tetramer: peptide: exchange factor = 50:5:1. The mixture was incubated in the dark at room temperature for 4 h. The peptide exchange efficiency was assessed by flow cytometry, with a replacement rate of ≥ 95% considered acceptable. Then the successfully exchanged peptide-HLA tetramer complexes were incubated with CTLs, followed by flow cytometric analysis to evaluate TCR binding. Unstained CTLs served as the blank control, while CTLs stained with a negative control peptide-loaded tetramer complex were used as the negative control.

### Enzyme-linked immunosorbent spot (ELISpot)

PBMCs and MCTLs were obtained from each patient as described above. IFN-*γ*–producing T cell numbers were visualized using anti-human IFN-γ ELISpot assay (Dakewe, China) according to the manufacturer’s protocol. The spots were counted and analyzed using the AID ELISpot plate reader software (Autoimmun Diagnostika).

### Statistical analysis

GraphPad Prism 8.0 (GraphPad Software, Inc., La Jolla, CA) was used for statistical analysis, and results are expressed as the mean ± standard deviation (SD). Student’s *t*-test was applied to compare the differences between two groups, and *p* < 0.05 was considered statistically significant. *p < 0.05; **p < 0.01; ***p < 0.001.

## Results

1. Patients

From June 2019 to August 2021, twenty-one patients were enrolled in this open-label, single-arm phase Ib clinical trial and received at least 1 cycle of MCTL/αPD-1 treatment. All the 21 patients enrolled were pathologically confirmed with NSCLC, the median age of the entire population (47.6% female, 52.4% male) was 59 years (range: 36–74 years). The clinical characteristics of the patients are listed in Table 1. The treatment details and entire management process of MCTL generated by personalized peptides-pulsed DC combined with Toripalimab therapy, including drug administration regimens and blood collection protocols are shown in Fig. 1.

**Table 1:** Clinicopathological characteristics of twenty-one advanced NSCLC patients

Characters	*N* = 21
*Age, y*	
≤ 60	12
> 60	9
*Gender*	
Male	11
Female	10
*ECOG performance status*	
0	7
1	13
2	1
*Smoking*	
Yes	13
No	7
Unknown	1
*Smoking index*	
≤ 400	0
> 400	13
*Pathology*	
Squamous	7
Adenocarcinoma	14
*Stage*	
IIIB	3
IV	18
*First-line regime*	
PTX + CBP/DDP	3
PEM + CBP/DDP	7
GEM + CBP/DDP	2
Others	9

2. Clinical efficacy

**Fig. 2:**
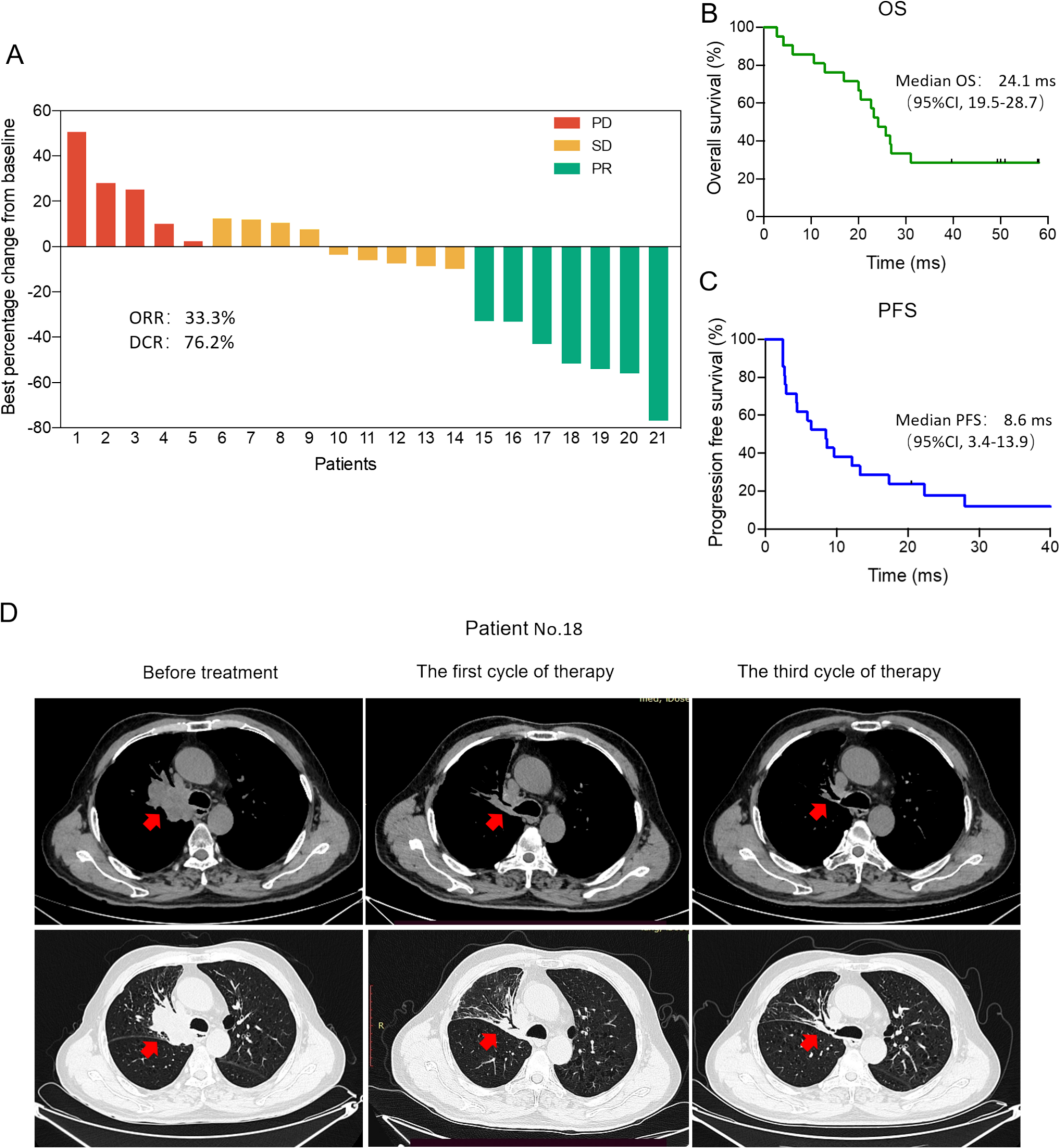
Clinical response of MCTL combined with Toripalimab. **A** Best percentage change from baseline in tumor lesions following the MCTL/Toripalimab therapy. **B** The OS and **C** PFS in patients received MCTL/Toripalimab therapy. **D** Chest CT images of the representative patient showing PR to the MCTL/ Toripalimab treatment. OS, overall survival; PFS, progression-free survival; ms, months; PR, partial response; SD, stable disease; PD, progressive disease; MCTL, multiple target cytotoxic T lymphocyte; ORR, objective response rate; DCR, disease control rate

Among the 21 patients, 7 have achieved PR, but unfortunately no patients had observed to evaluated CR, the ORR for patients treated with MCTL combined with Toripalimab was 7/21 (33.3%), showing an obvious increase than patients in checkmate 017 (20%) and checkmate 057 (19%), other second-line clinical studies for lung cancer. The DCR in this study was 16/21 (76.2%). The best percentage change in tumor measurement from baseline in target lesions is shown in Fig. 2A. As for the survival outcomes, at treatment assessment cutoff (June 2024), the median follow-up duration was 38.5 months, and the median OS (mOS) was 24.1 months (95% CI: 19.5 to 28.7 months), and 1 year and 2 year OS rates was 81% and 52.4% respectively (Fig. 2B). The median PFS (mPFS) was 8.6 months (95% CI, 3.4 ms to 13.9 ms), and 1 year PFS rate was 41.9% (Fig. 2C). Figure 2D shows representative pre- and post-treatment chest CT images from a patient (patient No. 18) who received two cycles of MCTL therapy combined with Toripalimab. After two cycles of MCTL/Toripalimab therapy, the patient demonstrated significant shrinkage of pulmonary tumor lesions, with short-term efficacy evaluated as PR.

3. Adverse events

An overview of all AEs occurring during the treatment was summarized in Table 2. The most commonly reported treatment-emergent AEs included hypothyroidism, hypophysitis, adrenal hypofunction and rash. All recorded AEs were grade 1 and no severe (grade 3–4) AEs or treatment-related deaths were observed. No clinical signs of autoimmune disorders or treatment-related diseases were identified during the clinical trial. Overall, the administered immunotherapy was well tolerated, and the safety profile remained favorable.

**Table 2:** Summary of treatment-emergent adverse events during MCTL/Toripalimab therapy

Adverse Events	Patients (%)	Grade
Hypothyroidism	3 (14.3)	1
Hypophysitis	1 (4.8)	1
Adrenal hypofunction	1 (4.8)	1
Rash	1 (4.8)	1

4. Immunological response

**Fig. 3:**
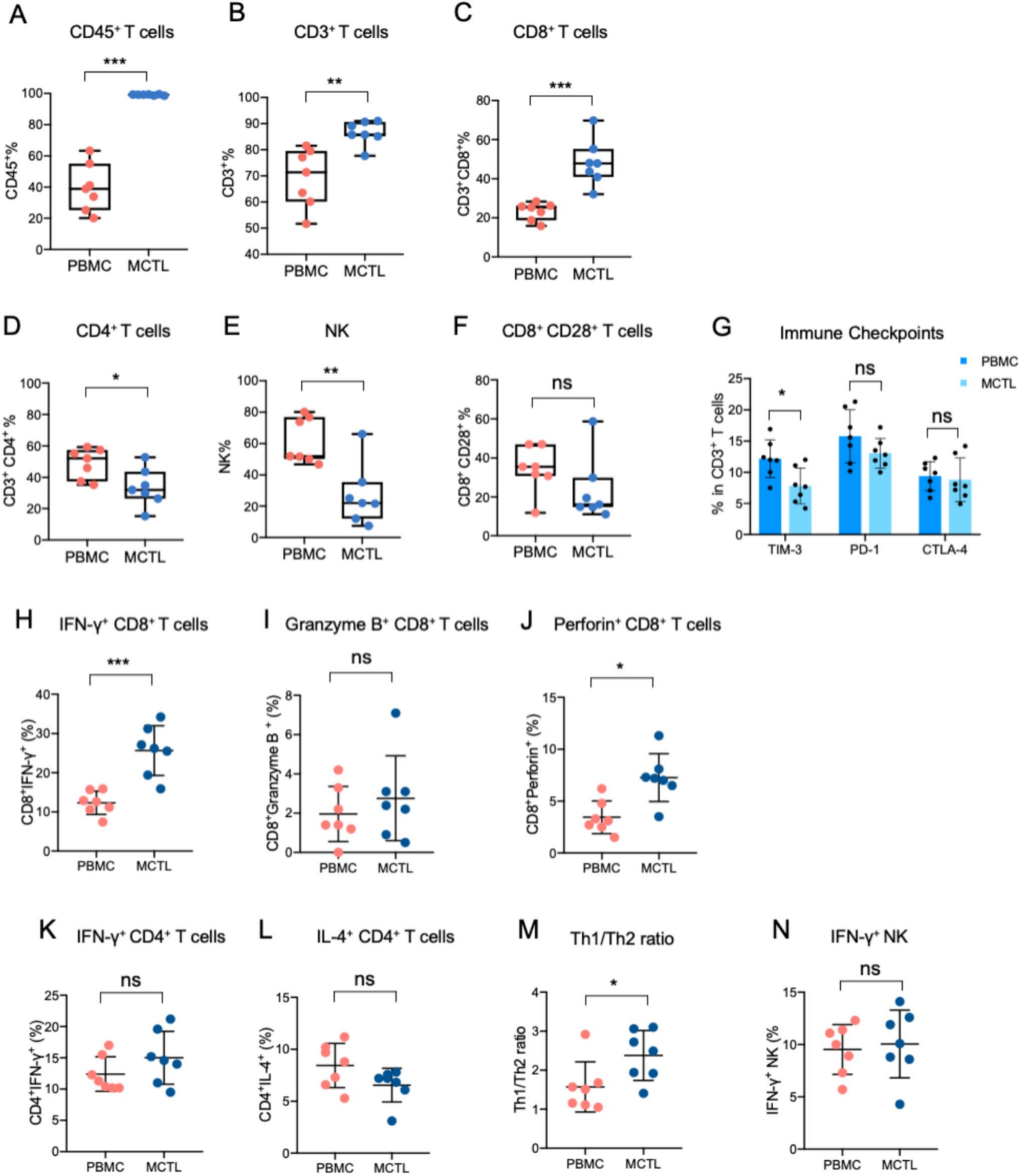
Important immune cell subsets and functions between MCTL and PBMC. **A**-**F** Frequencies of important immune cell subsets between MCTL and PBMC. **G** Important immune checkpoints expression on CD3 ^+^ lymphocytes between MCTL and PBMC. Cytokine production of CD8 ^+^ lymphocytes **H**-**J**, CD4 ^+^ lymphocytes **K**-**M** and NK cells **N** between MCTL and PBMC. MCTL, multiple target cytotoxic T lymphocyte; PBMC, peripheral blood mononuclear cell; NK, natural killer; Th, T helper. Data are presented as mean ± SD. *p < 0.05; **p < 0.01; ***p < 0.001; ns, not significant. Unpaired two-tailed Student’s *t*-test.

Immune cell subsets and functions within MCTLs were analyzed using flow cytometry. Compared with PBMCs extracted from the same patient, it showed a significant increase in important immune cells, like CD45^+^leukocytes, CD3^+^T cells, and CD8^+^T cells in MCTL (Fig. 3A-C), but for CD4^+^T cells and NK cells, these two subsets decreased in MCTL (Fig. 3D-E). Unfortunately, the frequency of activated T cells (CD28^+^CD8^+^T cells) remains unchanged (Fig. 3F), while we tested the common immune checkpoints expression in CD3^+^T cells, it was found that the levels of TIM-3 were downregulated in the CD3^+^T cells in MCTL than that in PBMC, the changes were significant (3G). Further investigation of cytotoxic lymphocytes demonstrated a marked increase in IFN-γ^+^CD8^+^ and Perforin^+^CD8^+^T cells in MCTL relative to PBMC, but there was no significance of Granzyme B expression in CD8^+^T cells (Fig. 3H-J). As for CD4^+^CTL (Fig. 3K-M), the production of IFN-γ and IL-4 in CD4^+^T cells remained stable; however, the Th1/Th2 ration elevated in the MCTL. The cytotoxic activity of NK cells was also evaluated but showed no differences between MCTL and PBMC (Fig. 3N).

**Fig. 4:**
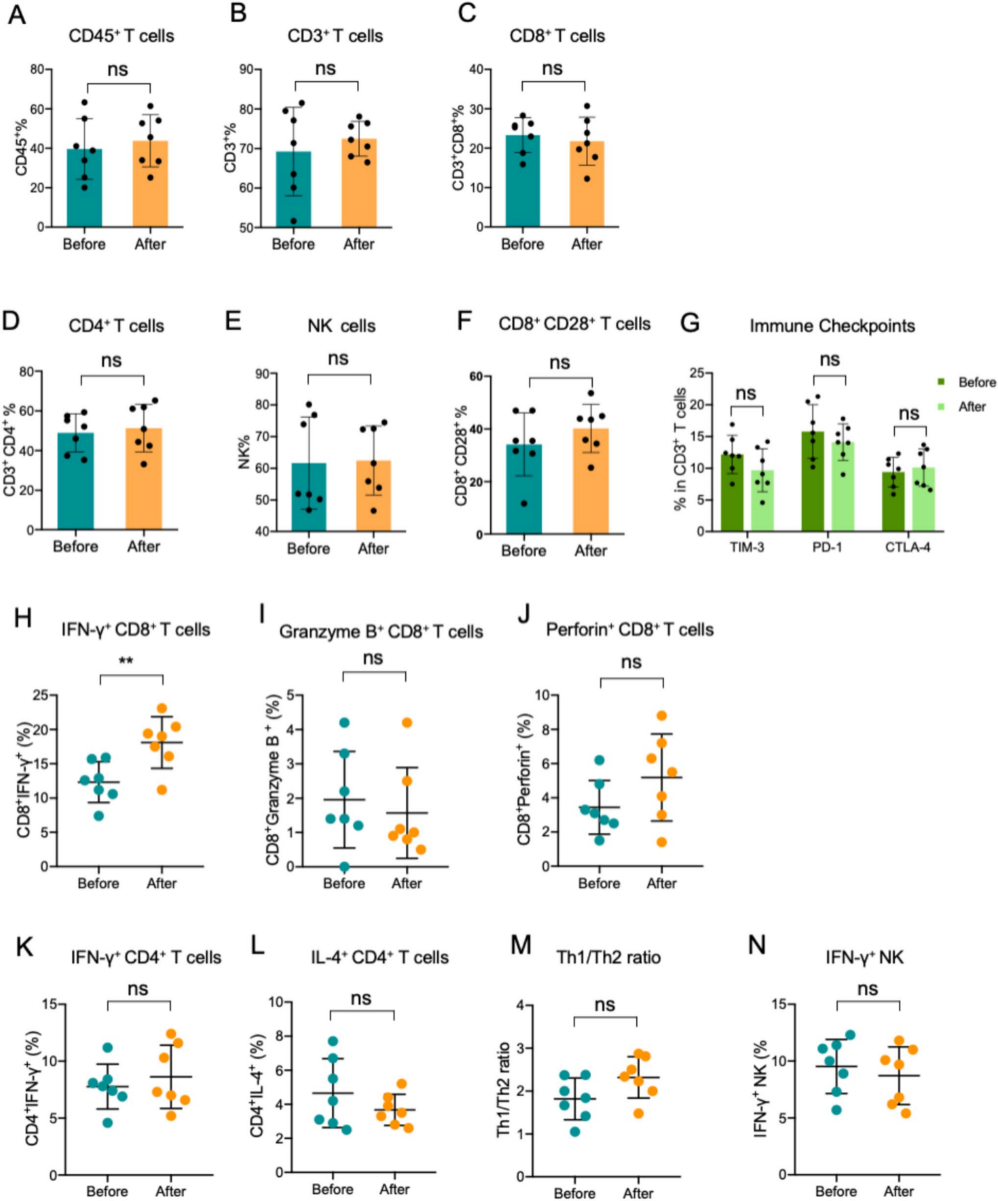
Important immune cell subsets and functions of patients before and one month after MCTL/Toripalimab therapy. **A**-**F** Frequencies of important immune cell subsets of patients before and after MCTL/Toripalimab therapy. **G** Important immune checkpoints expression on CD3 + lymphocytes of patients before and after MCTL/Toripalimab therapy. Cytokine production of CD8 + T lymphocytes **H**-**J**, CD4 + T lymphocytes **K**-**M** and NK cells **N** of patients before and after MCTL/Toripalimab therapy. MCTL, multiple target cytotoxic T lymphocyte; PBMC, peripheral blood mononuclear cell; NK, natural killer; Th, T helper. Data are presented as mean ± SD. **p < 0.01; ns, not significant. Unpaired two-tailed Student’s *t*-test

To further assess changes in immune status and immune cell subsets, functional parameters were evaluated in PBMC samples collected before treatment and one month after MCTL infusion. No statistically significant alterations were noted in major immune cellular subsets following therapy (Fig. 4A-F). Similarly, the immune checkpoints expression in CD3^+^T cells remained unchanged post-treatment (Fig. 4G). To verify the immune function of these immune cells, the expression levels of cytokines were detected in PBMC by flow cytometry. After treatment, the CD8^+^T cell function was improved with a significant higher IFN-γ secretion (Figs. 4H), but other cytokines did not show significant alterations (Figs. 4I-N), which indicated that CD8^+^T cells had a more potent cytotoxic function after transfusion. These findings suggest that MCTL combined with anti-PD-1 therapy can transiently enhance CD8^+^T cell-mediated immune function.

Furthermore, IFN-*γ* ELISPOT assay was performed to assess the T cell specific antigen peptides. MCTL and PBMC samples collected one month after MCTL infusion were analyzed. The IFN-γ spot counts were presented in Figure S1A. The results demonstrated that the number of IFN-γ spots generated by MCTL was significantly higher than that by PBMC, which exhibited that the MCTL have a relatively high ability to induce activated tumor-specific CTLs. For individual antigens for each patient, ELISPOT assays were also conducted for individual antigens in each patient, with results shown in Figure S1B. There is considerable interpatient variability in IFN-*γ* production capacity of specific antigen-specific T cells, which reflects, to some extent, differences in immune response status among patients. The MCTL from patient 6 seems to be more responsive to tumor-specific antigen peptides than PBMC; however, patient 6 suffered a disease progression and had a shorter survival (OS = 10.57 ms), These findings suggest that cellular IFN-*γ* production may not directly correlate with disease progression or survival.

5. Influence on therapeutic outcomes

**Fig. 5:**
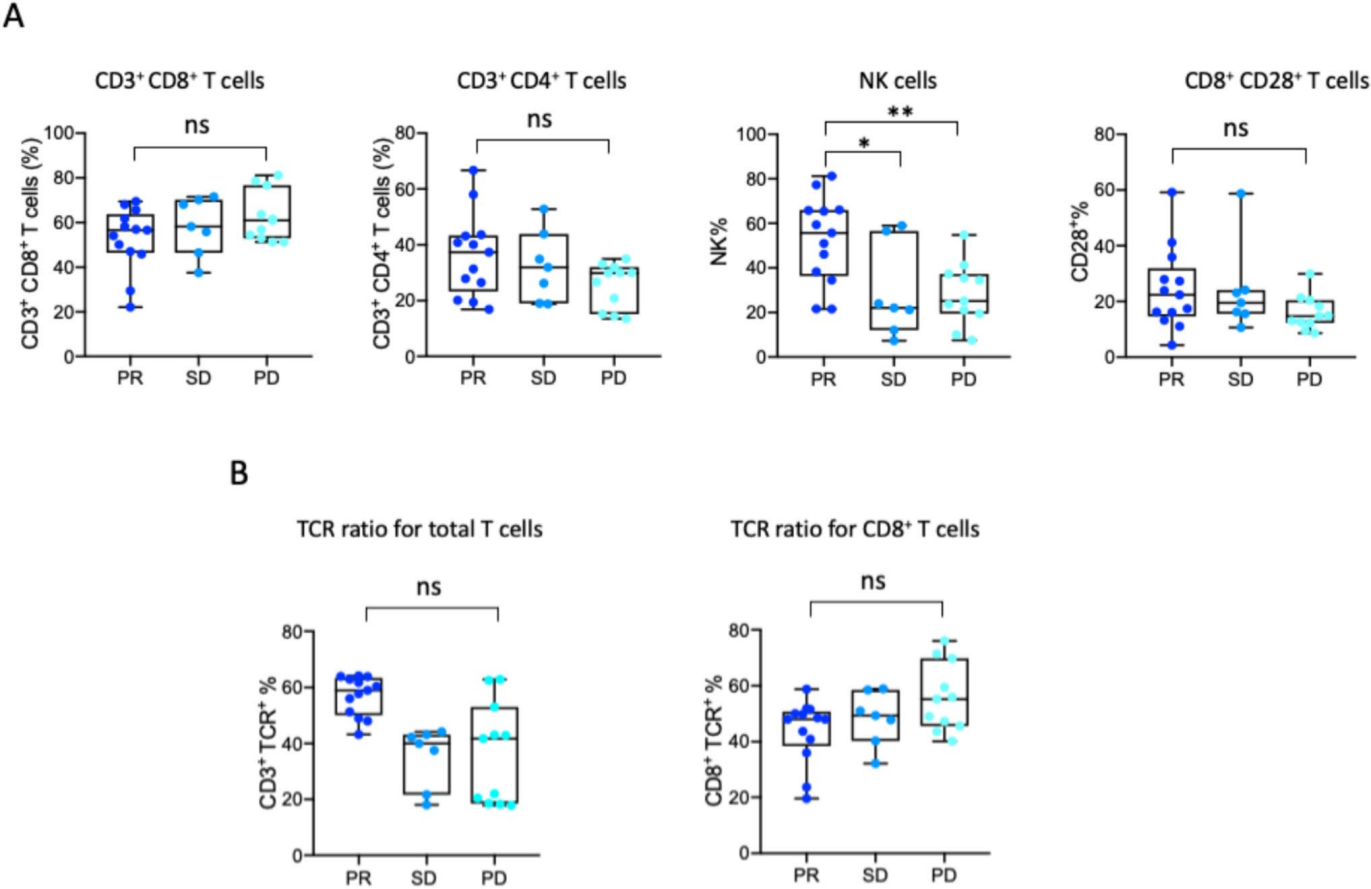
Important immune cell subsets and TCR ratios in MCTL across different clinical response groups. **A** The immune cell subsets in patients with different clinical responses following MCTL/Toripalimab therapy. **B** TCR ratios in total T cells and CD8⁺ T cells in patients with different clinical responses following MCTL/Toripalimab therapy. MCTL, multiple target cytotoxic T lymphocyte; TCR, T cell receptor; PR, partial response; SD, stable disease; PD, progressive disease; NK, natural killer. Data are presented as mean ± SD. *p < 0.05; **p < 0.01; ns, not significant. Unpaired two-tailed Student’s *t*-test

We next investigated the presence of immunological response that could correlate with therapeutic efficacy and survival of patients. Results showed that the frequencies of immune cell subsets (CD8^+^/CD4^+^/CD28^+^CD8^+^) in MCTL demonstrated no significant relationship with short-term clinical response in patients (Fig. 5A). Interestingly, patients with a higher proportion of NK cells tended to exhibit better short-term efficacy, given that NK cells constitute a minor proportion in MCTL and their frequency shows no significant difference compared to PBMC, it is unlikely that NK cells could serve as a short-term prognostic biomarker for MCTL/PD-1 therapy.

We next stratified patients into two comparative groups (PR + SD vs. PD, or PR vs. SD + PD) to evaluate the influence on short-term therapeutic outcomes. As shown in Figure S2, aside from a significantly higher proportion of CD4⁺T cells in the PR + SD group compared to PD, the remaining immunological parameters were generally consistent with the findings described above.

We next analyzed the tumor antigen–specific TCR expression ratio within MCTL to determine whether the proportion of tumor antigen–specific TCR-positive T cells was associated with patients’ therapeutic efficacy. The results indicated no significant differences in total TCR ratio or CD8^+^TCR ratio between patients with differing short-term treatment responses (Fig. 5B), suggesting that TCR ratio is not associated with short-term therapeutic efficacy.

**Fig. 6:**
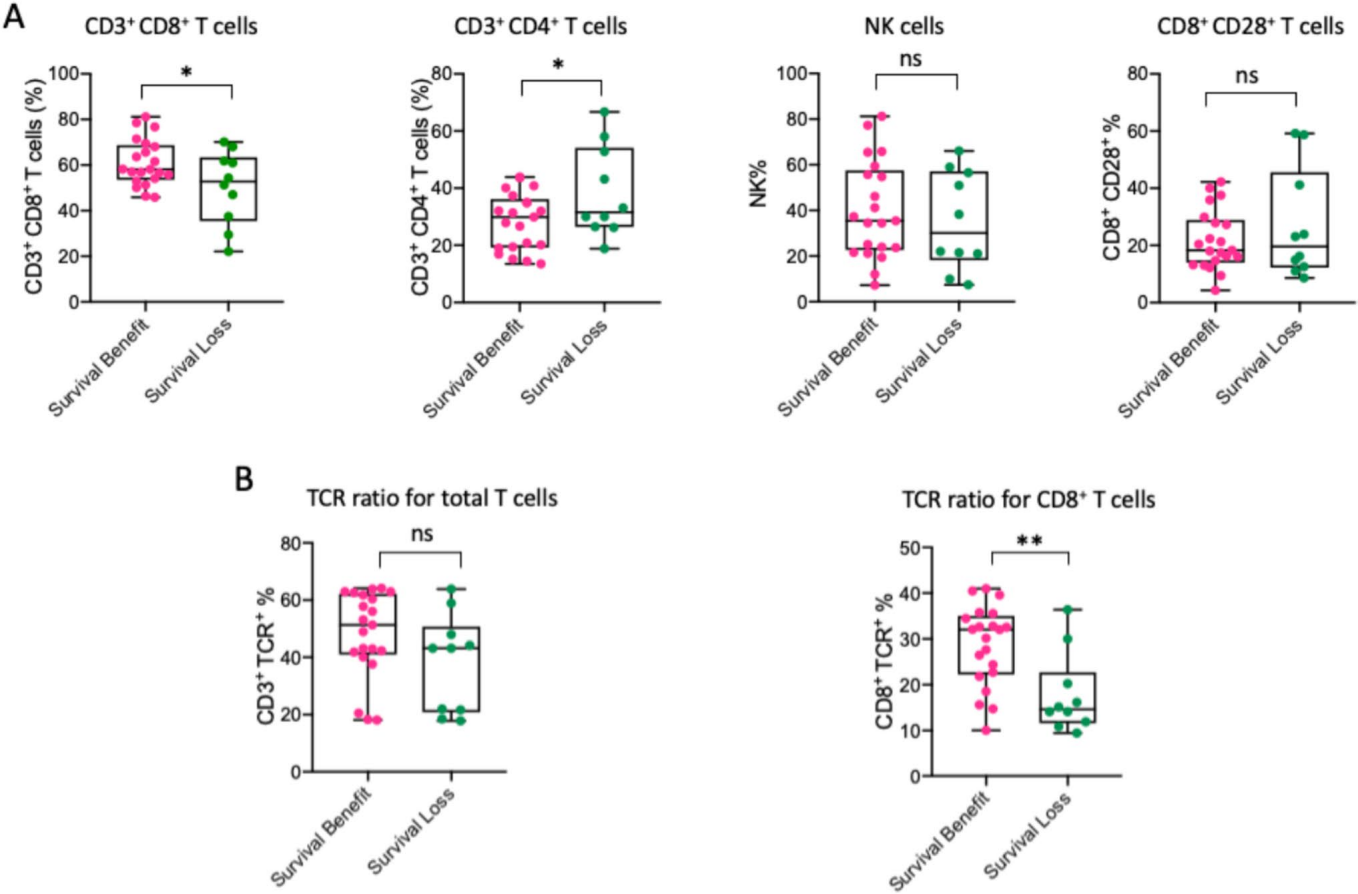
Impact of important immune cell subtypes in MCTL different survival outcomes. **A** The immune cell subsets in MCTL among patients with different survival outcomes following MCTL/Toripalimab therapy. **B** TCR ratios in total T cells and CD8⁺ T cells in MCTL among patients with different survival outcomes following MCTL/Toripalimab therapy. MCTL, multiple target cytotoxic T lymphocyte; TCR, T cell receptor; NK, natural killer. Data are presented as mean ± SD. *p < 0.05; **p < 0.01; ns, not significant. Unpaired two-tailed Student’s *t*-test.

Additionally, we then investigated how the immunological responses influence the patients’ survival. Patients were stratified into two groups based on whether their overall survival was above or below the median OS (24.1 ms), respectively. Patients in whom the OS was shorter than the median OS were considered to have survival loss, and patients in whom the OS was longer than the median OS were considered to have survival benefit. The data indicated that compared with patients who experienced survival loss, patients who got a survival benefit has a huge elevation in CD8^+^ and a decrease in CD4^+^T cells in MCTL (Fig. 6A), although no differences were observed in NK cells or CD8^+^CD28^+^T cells. Regarding TCR ratios, CD8^+^TCR ratio corresponded with the CD8^+^T cells percentage findings, such that patients with higher CD8^+^TCR ratio tended to have better survival outcomes (Fig. 6B).

**Fig. 7:**
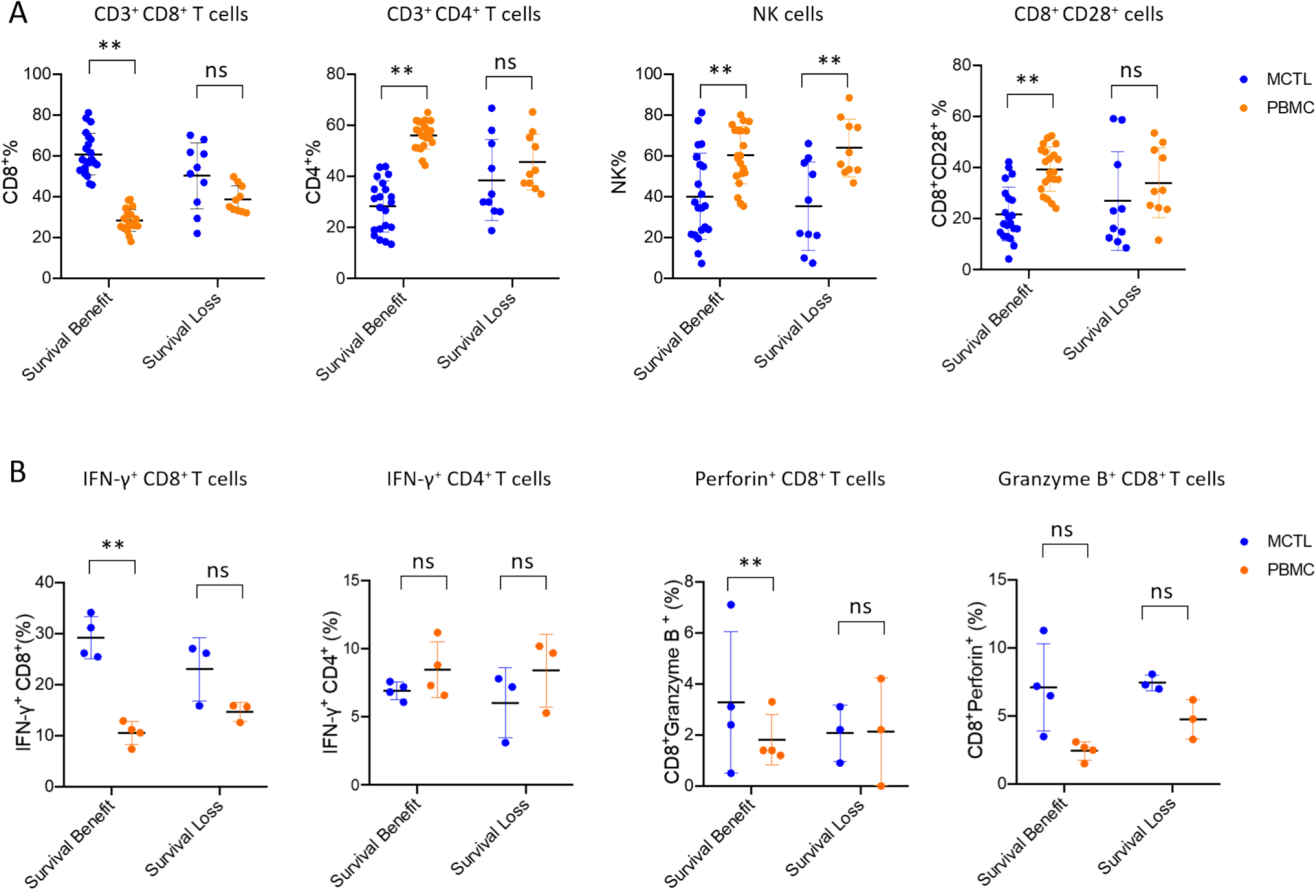
Immune cell subtypes and functions in MCTL and PBMC across different survival outcomes. **A** The immune cell subsets in MCTL and PBMC from patients with different survival outcomes following MCTL/Toripalimab therapy. **B** Cytokine production by CD8 ^+^ and CD4 ^+^ T cells in MCTL and PBMC from patients with different survival outcomes following MCTL/toripalimab therapy. MCTL, multiple target cytotoxic T lymphocyte; PBMC, peripheral blood mononuclear cell; NK, natural killer. Data are presented as mean ± SD. *p < 0.05; **p < 0.01; ns, not significant. Unpaired two-tailed Student’s *t*-test.

Interestingly, we found that compared to patients with survival loss, those who achieved survival benefits not only exhibited higher frequencies of CD8^+^T cells and CD8^+^TCR but also demonstrated a more pronounced disparity in the CD8^+^T cell ratio between MCTL and PBMC (Fig. 7A). Furthermore, CD4^+^T cells, NK cells, and CD8^+^CD28^+^cells followed an opposite trend to CD8^+^T cells, suggesting that patients with increased CD8^+^T cells and decreased CD4^+^T cells in MCTL relative to PBMC may have better survival outcomes (Fig. 7A). We further investigate the CD8^+^T cells functions between different survival group, results showed that MCTL has a marked increase in IFN-γ^+^CD8^+^ and Perforin^+^CD8^+^T cells than PBMC in patients who achieved survival benefit than those who experience survival loss, but there was no significance of Granzyme B expression in CD8^+^T cells (Fig. 7B). As for CD4^+^CTL (Fig. 7B), the production of IFN-*γ* in CD4^+^T cells remained stable. These findings suggest that the composition of CD8^+^T cell subsets and their cytotoxic functions within MCTL may influence patient survival, rather than short-term treatment response.

## Discussion

Neoantigens arise from mutations in cancer cells and are critical targets for T cell-mediated immunity, playing a pivotal role in eliciting effective antitumor responses [12–14]. The MCTL therapy is a novel neoantigen-specific ACT therapy based on the MCTL^®^ Cluster Target Peptide Screening and Identification Database (patent CN 104655849B). By performing high-throughput screening of antigenic peptides from the patient’s peripheral blood, the detected peptide sequences are compared with this database to construct a personalized cluster of tumor-specific antigen peptides. These peptides are then loaded onto DCs and co-cultured with cytokine-induced killer (CIK) cells to induce and expand a population of CTLs with multi-target recognition capability. This approach enables dynamic monitoring of changes in a patient’s antigenic peptide profile and helps to overcome tumor immune evasion, thereby endowing MCTL with theoretically more precise and durable antitumor activity.

Immunotherapy with PD-1/PD-L1 inhibitors has revolutionized the treatment landscape of NSCLC, providing durable survival benefits for a subset of patients [4]. However, most patients eventually experience disease progression, underscoring the need for novel therapeutic strategies to enhance immune efficacy. Recent studies suggest that combining neoantigen-specific ACT with ICIs can overcome immune suppression and induce durable antitumor responses, achieving superior efficacy compared with ICIs alone [15]. Therefore, combining MCTL with PD-1 blockade may simultaneously counteract antigen loss and immune suppression, thereby improving long-term clinical outcomes.

In our study, the combination of MCTL with Toripalimab in the second-line setting achieved an ORR of 33.3%, a DCR of 76.2%, a mPFS of 8.6 months, and a mOS of 24.1 months. For patients with advanced NSCLC progressing after first-line therapy, standard second-line options include docetaxel, pemetrexed, gemcitabine, or docetaxel combined with anti-angiogenic agents such as nintedanib or ramucirumab. Historically, docetaxel monotherapy achieved an ORR of 7.1%–24% and a mOS of approximately 7 months [16]. Combining chemotherapy with anti-angiogenic agents further improved outcomes: in LUME-Lung1, docetaxel plus nintedanib achieved an mPFS of 4.4 months and a mOS of 12.6 months, while the REVEL study reported a mPFS of 4.5 months and a mOS of 10.5 months with docetaxel plus ramucirumab [17, 18].

The CheckMate 017 [19] and CheckMate 057 [20] trials evaluated nivolumab monotherapy versus docetaxel in patients with previously treated squamous and non-squamous NSCLC, respectively. Nivolumab achieved ORRs of 20 and 19% with mOS of 9.2 and 12.2 months, respectively, both significantly superior to docetaxel. These pivotal trials established PD-1 inhibition as the standard second-line therapy for advanced NSCLC. The Lung-MAP S1800A trial evaluated pembrolizumab plus ramucirumab, reporting an ORR of 28%, a mPFS of 4.5 months, and a mOS of 14.5 months [21].

Beyond chemotherapy, anti-angiogenic regimens, and immune checkpoint inhibitors, Lifileucel (LN-145)—an autologous tumor-infiltrating lymphocyte (TIL) therapy—demonstrated promising efficacy in ICIs—resistant NSCLC in a Phase II trial, achieving an ORR of 21.4% [22].

Compared with these results, our MCTL plus Toripalimab regimen achieved a markedly prolonged mOS of 24.1 months and durable responses, suggesting enhanced immune activation and tumor-microenvironment re-sensitization in previously treated NSCLC.

Repeated long-term administration of MCTL was well tolerated, with no severe toxicities observed. The most frequently reported AEs was hypothyroidism. Notably, hypothyroidism has also been documented as the most common adverse event associated with Toripalimab [23]. Based on these observations, we hypothesize that MCTL may exhibit a more favorable safety profile.

T lymphocytes can be activated by tumor antigen-loaded DC and differentiate into CTL, exert tumor-killing effect [24]. In this study, DC were stimulated with multiple personalized tumor antigen peptides and presented the antigen peptides to the T lymphocytes, converting them into MCTL. Following reinfusion, the MCTL elicit potent tumor-killing activity via the production of multiple immunomodulatory cytokines like IFN-γ and Perforin. The cytokines, particularly IFN-*γ*, are recognized as critical immunomodulatory factors that play a pivotal role in tumor cell killing and shaping the tumor immune microenvironment. Our results demonstrated significantly higher proportions of IFN-γ^+^CD8^+^ and Perforin^+^CD8^+^T cells and an elevated Th1/Th2 ratio in MCTL compared with PBMCs, underscoring its robust immune activation capacity. Furthermore, compared to PBMC before MCTL/αPD-1 treatment, post-treatment PBMC also exhibited a significant increase in IFN-γ^+^CD8^+^T cells. This indicates that MCTL not only possesses intrinsic antitumor activity but also modulates and reshapes the tumor immune microenvironment, thereby enhancing the antitumor response of conventional PBMC.

Nevertheless, as this phase Ib study was an exploratory, single-arm trial primarily designed to evaluate the safety and feasibility of MCTL therapy, it has inherent limitations, including a relatively small sample size and the absence of a randomized control arm. A subsequent multicenter phase II randomized controlled trial is planned to further validate the efficacy and safety of MCTL therapy.

## Conclusions

In summary, the combination of MCTL with Toripalimab demonstrated encouraging clinical efficacy and durable responses with an excellent safety profile. Our findings suggest that the integration of MCTL/ Toripalimab therapy can manifest antitumor activity and extend survival, thereby offering a promising personalized treatment approach for NSCLC.

## Supplementary Information

Below is the link to the electronic supplementary material.

Supplementary file1 (DOCX 5592 KB)

## Data Availability

The datasets used and/or analyzed during the current study are available from the corresponding author on reasonable request.
