# Supplementary material for: A single-arm phase Ib study of personalized peptide-pulsed dendritic cell-based multiple target cytotoxic T lymphocyte immunotherapy in combination with toripalimab as second-line therapy in advanced non-small-cell lung cancer
Source: Cancer Immunol Immunother. 2026 Mar 11;75(4):102. doi: 10.1007/s00262-026-04338-7 (PMC12979723; doi:10.1007/s00262-026-04338-7)
Supplement: Supplementary file 1 — Supplementary file1 (DOCX 5592 KB) [file 262_2026_4338_MOESM1_ESM.docx]

**Supplementary materials**


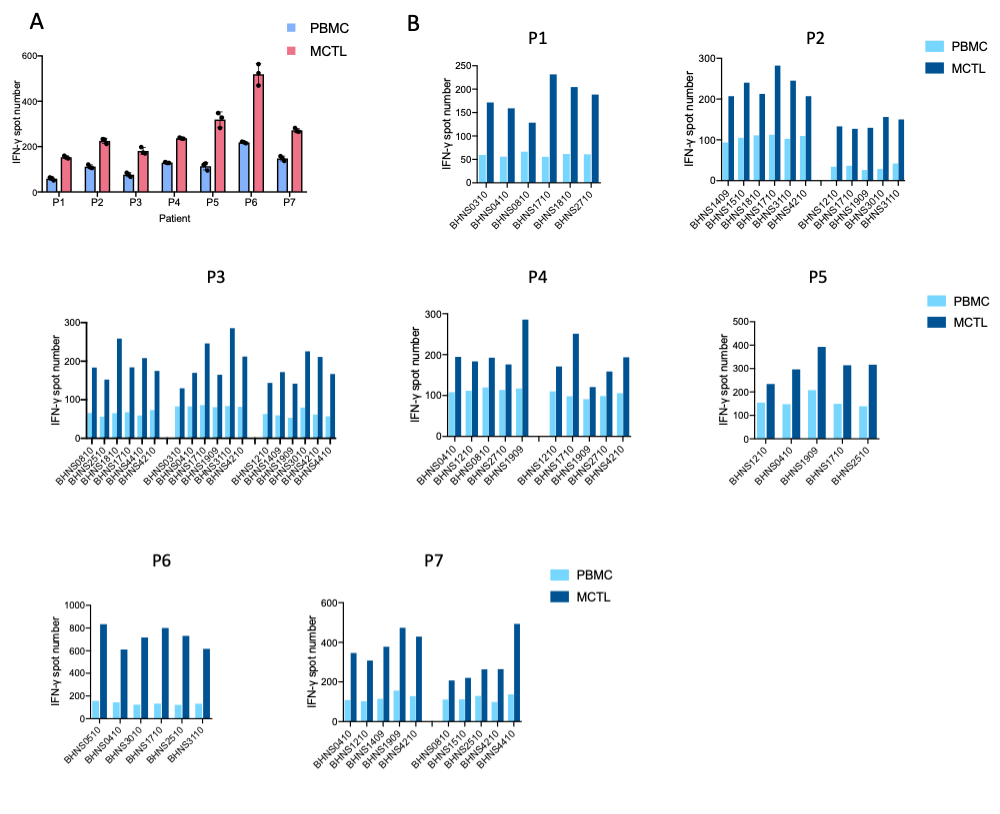


Figure S1: IFN-γ production detected by ELISPOT assay in 7 patients stimulated with different individual antigenic peptides. (A) IFN-γ spot number in 7 patients for the total mixed antigenic peptides. (B) IFN-γ spot number in 7 patients with different individual antigenic peptides. P, patient. MCTL, multiple target cytotoxic T lymphocyte; PBMC, peripheral blood mononuclear cell.


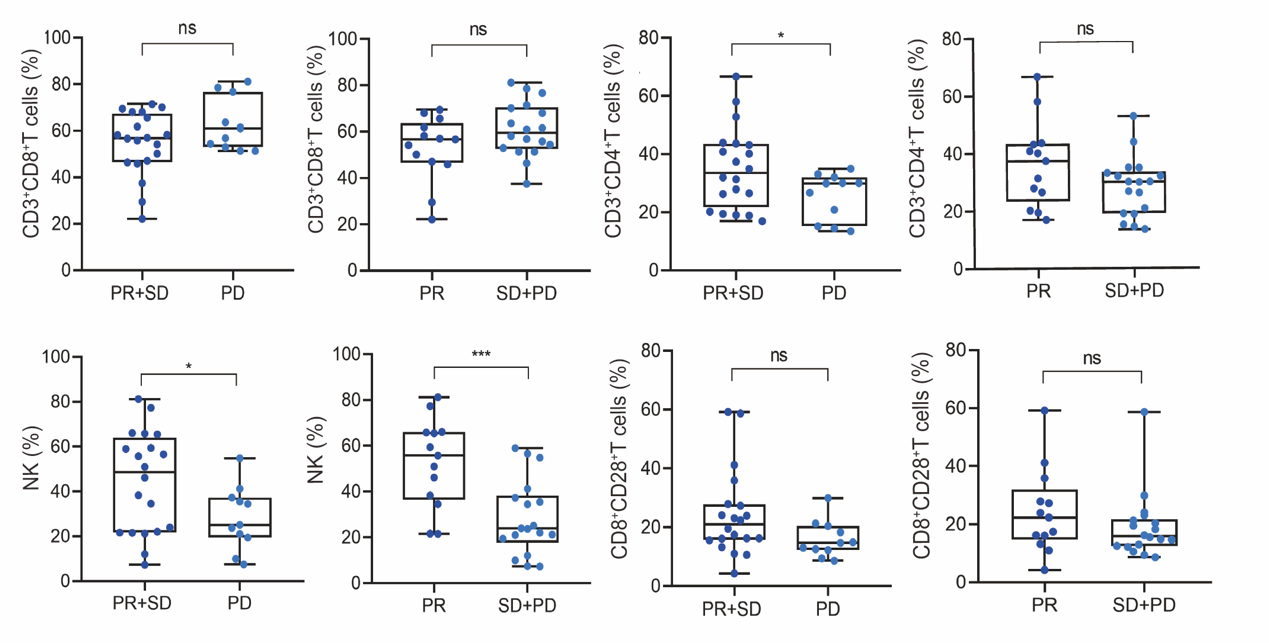


Figure S2: The important immune cell subsets across different clinical response groups (PR+SD vs. PD, or PR vs. SD+PD). PR, partial response; SD, stable disease; PD, progressive disease; NK, natural killer. Data are presented as mean ± SD. *p < 0.05; **p < 0.01; ns, not significant. Unpaired two-tailed Student’s t-test.

Table S1: The specific antigen peptides for every single patient

| No. of Patient | ID of antigen peptides | Total number of antigen peptides |
| --- | --- | --- |
| 1 | BHNS3110, BHNS0810, BHNS4410, BHNS0710, BHNS3510, BHNS0310, BHNS0110, BHNS0609, BHNS1610, BHNS1210, BHNS1409, BHNS1510, BHNS0710 | 13 |
| 2 | BHNS4010, BHNS3209, BHNS3209, BHNS3010, BHNS2210 | 5 |
| 3 | BHNS0110, BHNS0710, BHNS1010, BHNS0609, BHNS2810, BHNS0810, BHNS3110, BHNS1409, BHNS2810, BHNS3310, BHNS4210, BHNS1710, BHNS1909, BHNS0510, BHNS2710, BHNS1610, BHNS0510, BHNS0609, BHNS0710, BHNS1409 | 20 |
| 4 | BHNS0110, BHNS1610, BHNS4010, BHNS4610, BHNS0609, BHNS1409, BHNS2810, BHNS0810, BHNS1710, BHNS1010, BHNS0310, BHNS1810, BHNS1410, BHNS0110, BHNS1409, BHNS1610 | 16 |
| 5 | BHNS1110, BHNS2710, BHNS4610, BHNS3010, BHNS0810, BHNS3110, BHNS3809, BHNS3310, BHNS0710, BHNS4010, BHNS0110, BHNS0609, BHNS0410, BHNS1909 | 14 |
| 6 | BHNS1710, BHNS2510, BHNS0710, BHNS0810, BHNS1810, BHNS4210, BHNS1909, BHNS3410, BHNS1409, BHNS3110, BHNS0410, BHNS2710 BHNS1210 | 13 |
| 7 | BHNS3110, BHNS0810, BHNS0710, BHNS2309, BHNS0410, BHNS1909, BHNS1710, BHNS2710, BHNS3609 | 9 |
| 8 | BHNS0210, BHNS1010, BHNS1710, BHNS3110, BHNS0110, BHNS0810, BHNS2309 | 7 |
| 9 | BHNS3110, BHNS2309, BHNS0710, BHNS0110, BHNS0810, BHNS2710 | 6 |
| 10 | BHNS2510, BHNS0810, BHNS0410, BHNS1710, BHNS4210, BHNS1909, BHNS0510, BHNS2710 | 8 |
| 11 | BHNS2810, BHNS1909, BHNS0710, BHNS4210, BHNS3110, BHNS1710, BHNS1810, BHNS0410, BHNS0810, BHNS0510 | 10 |
| 12 | BHNS0710, BHNS2810, BHNS4210, BHNS0510 | 4 |
| 13 | BHNS0710, BHNS4210, BHNS1710, BHNS0510, BHNS0810, BHNS2710 | 6 |
| 14 | BHNS1210, BHNS1909, BHNS3410, BHNS1710, BHNS2710, BHNS0710, BHNS0510 | 7 |
| 15 | BHNS1710, BHNS0410, BHNS2710, BHNS0310, BHNS1810, BHNS0810 | 6 |
| 16 | BHNS3110, BHNS1409, BHNS1710, BHNS1510, BHNS4210, BHNS1810, BHNS1210, BHNS3010, BHNS1909 | 9 |
| 17 | BHNS2510, BHNS4210, BHNS1710, BHNS1810, BHNS0810, BHNS4410, BHNS0310, BHNS0410, BHNS3110, BHNS1909, BHNS1210, BHNS1409, BHNS3010 | 13 |
| 18 | BHNS1210, BHNS2710, BHNS1909, BHNS0410, BHNS0810, HNS1710, BHNS4210 | 7 |
| 19 | BHNS2510, BHNS1210, BHNS1710, BHNS1909, BHNS0410 | 5 |
| 20 | BHNS2510, BHNS1710, BHNS3010, BHNS0510, BHNS0410, BHNS3110 | 6 |
| 21 | BHNS1409, BHNS1210, BHNS1909, BHNS0410, BHNS4210, BHNS2510, BHNS4410, BHNS0810, BHNS1510, BHNS3110, BHNS2710, BHNS0510 | 12 |
